# An In-Depth View of the Porcine Trabecular Meshwork Proteome

**DOI:** 10.3390/ijms20102526

**Published:** 2019-05-22

**Authors:** Sebastian Funke, Vanessa M. Beutgen, Lea Bechter, Carsten Schmelter, Vanessa Zurawski, Natarajan Perumal, Norbert Pfeiffer, Franz H. Grus

**Affiliations:** Experimental and Translational Ophthalmology, Department of Ophthalmology, University Medical Center, 55101 Mainz, Germany; sfunke1@gmx.de (S.F.); vbeutgen@eye-research.org (V.M.B.); lea.be@web.de (L.B.); cschmelter@eye-research.org (C.S.); vzurawsk@gmail.com (V.Z.); nperumal@eye-research.org (N.P.); norbert.pfeiffer@unimedizin-mainz.de (N.P.)

**Keywords:** trabecular meshwork, glaucoma, proteomics, mass spectrometry, model organism, *Sus scrofa*

## Abstract

The house swine (*Sus scrofa domestica* Linnaeus 1758) is an important model organism regarding the study of neurodegenerative diseases, especially ocular neuropathies such as glaucoma. This is due to the high comparability of the porcine and human eye regarding anatomy and molecular features. In the pathogenesis of glaucoma, the trabecular meshwork (TM) forms a key ocular component in terms of intraocular pressure (IOP) elevation. Thereby, functional TM abnormalities are correlated with distinct proteomic alterations. However, a detailed analysis of the TM proteome has not been realized so far. Since the porcine eye has high potential as a model system to study ocular diseases such as glaucoma, the present study focuses on the in-depth analysis of the porcine TM proteome. By use of a bottom-up (BU) mass spectrometric (MS) platform utilizing electrospray ionization liquid chromatography tandem MS (LC-ESI-MS/MS) considering database-dependent and peptide de novo sequencing, more than 3000 TM proteins were documented with high confidence (FDR < 1%). A distinct number of proteins with neuronal association were revealed. To the best to our knowledge, many of these protein species have not been reported for TM tissue before such as reelin, centlein and high abundant neuroblast differentiation-associated protein AHNAK (AHNAK). Thereby, AHNAK might play a superordinate role in the TM regarding proposed tissue involvement in barrier function. Also, a high number of secretory proteins could be identified. The generated TM proteomic landscape underlines a multifunctional character of the TM beyond representing a simple drainage system. Finally, the protein catalogue of the porcine TM provides an in-depth view of the TM molecular landscape and will serve as an important reference map in terms of glaucoma research utilizing porcine animal models, porcine TM tissues and/or cultured TM cells.

## 1. Introduction

The trabecular meshwork (TM) is a highly specialized eye tissue responsible for the regulation of the anterior eye chamber aqueous humor (AH) outflow and the control of the intraocular pressure (IOP) [1,2]. Since an elevated IOP represents the predominant risk factor for primary open angle glaucoma (POAG) in humans [3,4], TM tissue and disease-associated structural, molecular and functional alterations are important structures in glaucoma research. Pescosolido et al. (2012) [5], for instance, postulated that particular glycosaminoglycans undergo specific pathogenic structural changes in the TM tissue of glaucoma patients and may responsible for IOP elevation. However, distinctive proteomic alterations of the TM tissue were correlated with glaucomatous conditions as recently reviewed in detail by our group [6]. The contractile TM, which is located at the corneoscleral transition zone, consists of different cellular components encompassing endothelial, stem and smooth muscle cells as well as the extracellular matrix (ECM). All components are organized in a functional system comprising the Schlemm‘s canal [2,7,8,9], which is represented by a multiple vessel system in most non-primate mammals including the domestic pig [10]. Considering the anatomical ocular architecture and neuronal comparability of pigs and humans, the house swine (*Sus scrofa domestica* Linnaeus 1758) represents an attractive model organism, especially for the study of ocular neuropathies including glaucoma [11,12,13,14]. Thereby, an important advantage is the unproblematic availability of porcine ocular tissue material for the establishment of an ocular organ culture [15]. With regard to the limited TM tissue amount which can be obtained from a single eye ball, the easy accessibility of porcine material is highly advantageous to source appropriate protein amount for in-depth discovery and other proteomic profiling studies. Regarding TM morphology, in comparison with other non-primate species, the domestic pig TM shows the highest similarity to the human TM [10,16]. Since TM structural and functional changes are associated with aberrant proteomic alterations [6] only a few works have characterized the complex TM proteome with high sensitivity, and a detailed protein catalogue of the porcine TM is still missing. Accordingly, the aim of the present work was to analyze the TM proteome of an important study animal by use of an established “bottom up” high performance liquid chromatography tandem mass spectrometry (BULCMS) workflow and to provide a detailed protein map, which can serve as a reference in terms of ocular proteomics in future. Sensitive TM proteomic analysis should importantly contribute to the molecular understanding of this highly specialized tissue. Finally, in correspondence with research on anatomical features of the porcine eye [14], the present proteomic work should assist with the establishment of the house swine as a reliable model system organism for ocular diseases with a special focus on glaucoma on the molecular level.

## 2. Results

**Figure 1:**
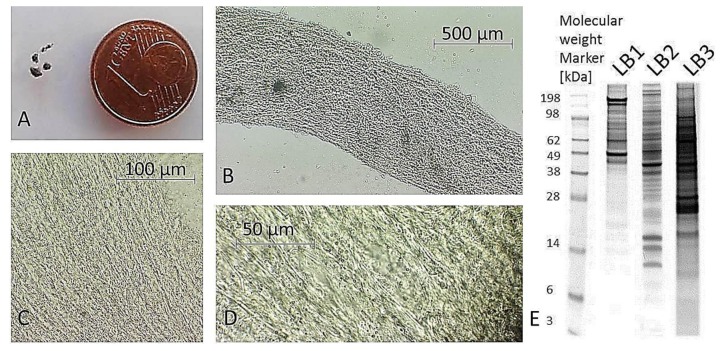
Processed porcine trabecular meshwork (TM). (**A**) TM sample acquisition is difficult due to small size of processed TM tissue as indicated by the coin comparison. (**B**) Isolated TM displays no connective tissue remains indicating the purity of TM tissue sample (5× magnification). TM sample shows a typical mesh-like structure ((**C**) 10× magnification, (**D**) 40× magnification), (**E**) TM corresponding exemplary extracts fractionated by 1D-SDS PAGE using three different lysis protocols (LB1 = 2 M thiourea, 7 M urea, 30 mM tris-HCl, 4% CHAPS, 2% ASB-14 (8 µg protein); LB2 = 50 mM tris-HCl, 8 mM Na_2_HPO_4_, 137 mM NaCl, 3 mM KCl, 0,5% SDS, 0,5% triton X-100, 0,5% DDM (8 µg protein); LB3 = 0.5% DDM (50 µg)); all different extracts were used for combinatory BULCMS TM proteome analysis.

**Figure 2:**
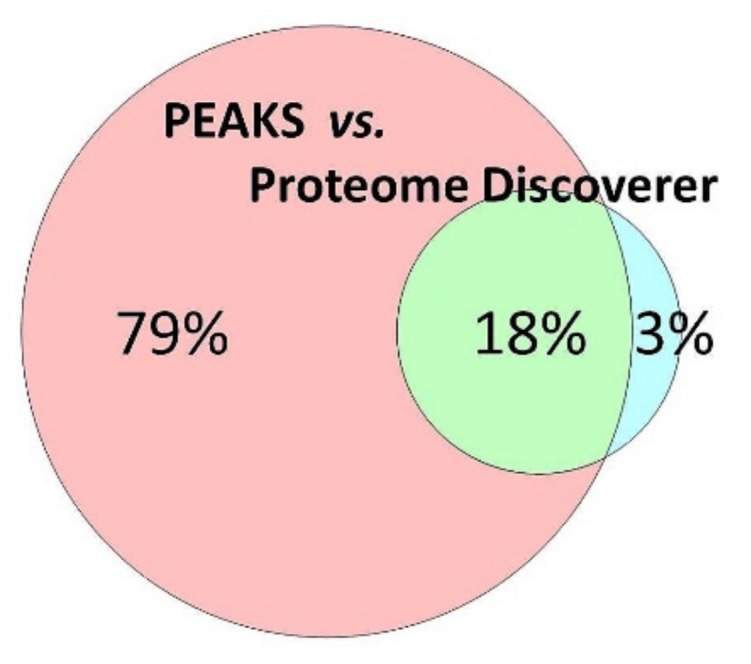
The majority of the porcine trabecular meshwork (TM) could be identified by use of the PEAKS combinatory de novo peptide sequencing/database related protein identification strategy. In summary, more 3747 proteins could be documented for the porcine TM.

**Figure 3:**
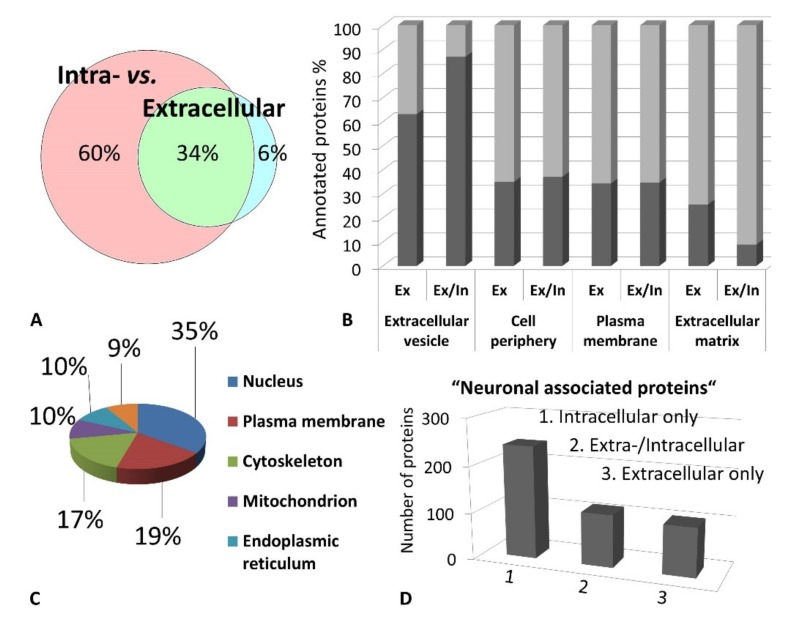
(**A**). The majority of identified proteins correspond to the intracellular fraction of the trabecular meshwork (TM) proteome inferred from gene ontology (GO) annotation analysis. (**B**) Exclusively extracellular proteins (Ex) as well as proteins with localization in the extra-and intracellular milieu (Ex/In) correspond predominantly to extracellular vesicles indicating secretory processes. (**C**) Most intracellular TM proteins correspond to the nucleus followed by the cellular plasma membrane. (**D**) The majority of “neuronal associated proteins” are intracellular protein species.

**Figure 4:**
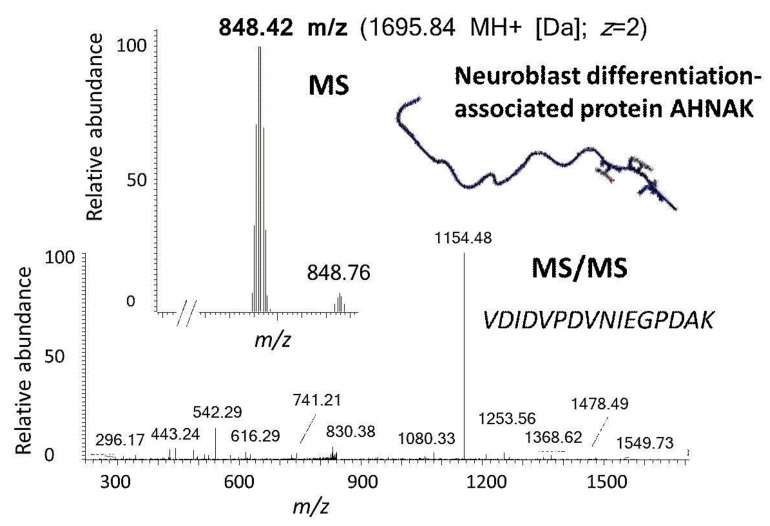
Exemplary identification of a prominent barrier function-related protein of neuronal relevance in the porcine trabecular meshwork (TM) by use of the established BULCMS workflow. A unique reporter peptide peak 848.42 *m/z* (MS) was fragmented by collision-induced decay (CID) (MS/MS) leading to the stringent identification of neuroblast differentiation-associated protein AHNAK (FDR < 1%) and was supported by both, database-related and de novo peptide sequencing analysis. (AHNAK image from the RCSB PDB (rcsb.org) of PDB ID 4FTG [22]).

Excised trabecular meshwork (TM) tissues showed a high degree of purity. No connective tissue contaminants could be indicated regarding the microscopic inspections (Figure 1A–D). TM extracts displayed distinctive 1D SDS PAGE protein patterns appropriate for bottom-up liquid chromatography-mass spectrometric (BULCMS) analysis (Figure 1E). BULCMS analysis resulted in the identification of more than 3000 proteins considering a high confident protein identification (FDR < 1%) (see Table S1). The highest number of identified proteins (79%) could be achieved by use of a combinatory database-related/de novo peptide sequencing strategy. Regarding the identified TM proteins a distinct degree of congruency could be achieved using both identification procedures (Figure 2). Approximately 93% of all identified proteins could be annotated to cellular components referring to GO analysis. The majority of proteins represented intracellular species, whereby only nearly 6% of annotated TM proteins could be exclusively associated with the extracellular milieu (Figure 3A). Thereby, besides the identification of collagens (I, III, IV), tenascins and laminins, further important extracellular matrix (ECM) proteins comprising lumican, nidigen-1 and 2, sushi nidogen and EGF-like domain-containing protein 1, γ, podocan, fibulin-5, retinoic acid receptor responder protein 2, calreticulin, EGF-containing fibulin-like ECM protein 1, prolyl 3-hydroxylase 1, biglycan, prolargin, matrix metalloprotease 9 and thrombospondins could be catalogued confidently with respect to the current literature concerning the composition of TM ECM subproteomes [17,18,19]. Also, contractile TM elements such as tropomyosin α and β, myosin 9, 10 and 11 as well as cytoskeletal proteins vimentin, actin and tubulin forms could be reported with high identification scores. In terms of the subcellular distribution, most annotated TM proteins were found to be associated with the nucleus (Figure 3C), e.g., histones, nucleolin, serine/arginine-rich splicing factor 3 and heterogeneous nuclear ribonucleoproteins A2/B1. Moreover, the detection of characteristic marker proteins expressed by TM cells including aquaporin-1, α-crystallin B chain, matrix Gla protein and smooth muscle actin [20] support the cellular representation within the whole TM tissue samples. Interestingly, retina-specific copper amine oxidase and further retinal proteins including rhodopsin, interphotoreceptor matrix proteoglycan 1, retinal dehydrogenase 1 and 2, retinaldehyde-binding protein 1 and 2, retinal guanylyl cyclase 2 and retinal-specific ATP-binding cassette transporter could be identified in the TM tissue samples. Moreover, two myelin proteins could be identified with scores indicating distinct abundance in the TM tissue: myelin protein P0 (Proteome Discoverer score = 1700; PEAKS − 10logP = 189) and myelin basic protein (Proteome Discoverer score = 113; PEAKS − 10logP = 67). Approximately 4% of all annotated proteins were found to be associated with the myelin sheet. Also, nearly 14% of the annotated TM proteins were found to be neuronal related (Figure 3D) with respect to the GO database categories “cellular component” and ”molecular function”, e.g., reelin, centlein, neuron navigator 3, neuroblastoma amplified sequence, neurobeaching-like proteins (1, 2), neurochondrin, neurofascin, neuron navigator 1 and neurabin-2 (see Table S2). Numerous neural marker proteins show moderate to high scores in correspondence with both protein identification software programs, e.g., neurofilament light polypeptide (ProtDisc = 1343, PEAKS − 10lgP = 100), glial fibrillary acidic protein (GFAP) (ProtDisc = 862, PEAKS − 10lgP = 154) or neural cell adhesion molecule 1 (ProtDisc = 150, PEAKS − 10lgP = 87), indicating high abundance of nerve-associated proteins in the TM tissues. Among the most abundant proteins with neuronal relevance, neuroblast differentiation-associated protein AHNAK (ProtDisc = 2537; PEAKS − 10lgP = 233) (Figure 4) could be recovered from TM tissues. Also, a high number of typical marker proteins associated with neuronal stress such as huntingtin and chaperones encircling heat shock proteins and crystallins could be detected. Furthermore, 46% of all TM proteins could be GO annotated regarding their molecular function. Also, approximately 11% of these annotated TM proteins were found to be involved in receptor binding including dopamine, type 2A serotonin, acetylcholine, GABA and glutamate receptor binding. Thereby, metabotropic glutamate receptors 1, 3 and 5 could be recovered from TM tissues. Ephrin type-B receptor 2, which is involved in axon guidance and translocator protein, are two exemplary important neuronal proteins recovered from TM tissues referred to in GO molecular function analysis. Furthermore, 13% of all annotated proteins are involved in cytoskeletal protein binding, especially actin binding. A comparison to the porcine retinal proteome [21] showed that approximately 47% of the focused porcine retina proteome was recovered in the TM tissue samples also comprising neuronal proteins. Approximately 66% of the TM proteins were exclusively documented for the TM proteome and have not been reported for the porcine retina proteome, which on the other hand underlines the ocular exclusivity of the TM proteome.

## 3. Discussion

The present work reports the largest trabecular meshwork (TM) protein catalogue to date, comprising more than 3000 highly confident TM-associated proteins. Moreover, extracellular as well as intracellular TM protein species could be recovered providing an in-depth view into the TM extracellular matrix (ECM) and TM cellular proteome of the house swine. Compared to former proteomic TM cell-related studies, a high congruency could be reported regarding studies cataloguing 235 [23], 718 [24], 853 [25] and 1644 proteins [26]. For the whole TM tissue specimen, 368 proteins have been recovered so far [27]. To our best knowledge, no study has been done on porcine TM tissues yet and therefore the protein map generated in the present work can serve as an important reference list in terms of ocular proteomics, especially suitable for glaucoma research using the domestic pig as a model organism. Based on similarities in architecture and molecular features the porcine eye has a high potential for the use as a model system in glaucoma [11,16] and recently our group provided a porcine retina organ culture for the study of glaucoma [15]. Regarding a comparison between the porcine retina and TM, numerous neuronal proteins are shared in both ocular sites; however, a high portion of TM proteins could be exclusively documented for the TM and has not been detected in the retina referring to a recent study [21], which might reflect the functional specialization but considering the neural origin. An important reason for the observed tissue specificity of the TM proteome most likely can be found in the TM ECM. Interestingly, TM ECM is highly comparative to the optic nerve head (ONH) lamina cribrosa ECM based on the proteomic level [28]. ECM and also its crosstalk with cell cytoskeleton play important roles in terms of the structural dynamic and the function of the TM [2]. A distinct recovery of TM ECM-specific proteins including tenascin (TNC), smooth muscle actin, fibronectin, integrins, collagen IV, laminin [1,29,30,31,32,33] support the proper illustration of the TM ECM proteome in the present work. Despite the fact that numerous ECM proteins are characteristic for TM ECM and neuronal ECM comprising laminins, collagens (I, IV), fibronectin and tenascin C [34], neurocan core protein, a neuronal specific ECM chrondoitin sulfate proteoglycan [35] was identified in the porcine TM in the present study. Also, the identification of many further neuronal-related proteins, such as highly abundant neuroblast differentiation-associated protein AHNAK in the TM is in confidence with the neural crest and the cranial paraxial mesodermal origin of the TM [36,37,38]. AHNAK is highly expressed in brain endothelial cells conciliating blood-brain barrier properties [39]. TM tissue-related barrier functions are important regarding the aqueous outflow channel isolation in the TM cribriform region with endothelial participation [8,40]. In light of this, it is highly thinkable, that referring to AHNAK TM tissue prominence, the protein plays a crucial role in maintaining this barrier function and therefore should be considered in future glaucoma studies with a special focus on TM. However, with respect to several neuronal proteins of lower abundance, their origin from TM surrounding retinal tissues might be assumed, and these proteins might represent tissue processing artifacts. Nevertheless, microscopic TM tissue quality inspection and high abundance of recovered key neuronal proteins, e.g., neurofilament polypeptides, indicate their TM-specific tissue origin. Also, traces of neuronal proteins could be sourced by scleral spur nerve endings suggested to function as afferent TM mechanoreceptors [41]. Interestingly, myelinated and unmyelinated nerves within the TM were supported for guinea pigs [42]. Also, innervation of the anterior eye outflow region was demonstrated in the house swine [43] and the presence of neuroregulatory cells was suggested for the primate TM [44]. Interestingly, TM cells display a similar CD44 cytotoxicity [45] such as neuroretinal cells, which supports the idea of a neuronal TM in accordance. This hypothesis is in confidence with the recovery of myelin basic, myelin protein P0 and further myelin associated proteins from the porcine TM tissues in the present study. Also, the detection of neuronal RNA binding proteins comprising RNA-binding protein Musashi homolog 1 and calcitonin [46] support the neuronal character of the TM tissue. Accordingly, TM proteomic data support the hypothesis of the TM-specific neuronal features. Despite the limitation to provide a subcellular proteomic view, but given the high number of recovered neural proteins, the present work contributes to the question of whether neural molecular features of the TM represent a contact point in glaucoma pathology. This thought is accompanied by the finding that comparable molecular events in TM and ONH drive neurodegeneration processes in glaucoma [47,48]. Regarding glaucoma related proteins, many of them could be identified in the porcine TM in this study, encompassing Toll-like receptor 4 [49], huntingtin [50], BMP-2-inducible protein [51], TGF-β-activated kinase 1 and MAP3K7-binding protein 1 [24], ADP/ATP translocase 3, heat shock proteins (e.g., HSP90β, HSP70) [52], crystallins [53,54,55,56] and integrins [57]. Also, retinol-binding protein 3, retinal dehydrogenase 2 and retinoic acid receptor responder protein 2 could be identified, which is interesting since TM expression of glaucoma-related myocilin [58] was demonstrated to be regulated by retinoic acid [59]. The recovery of glaucoma-associated proteins from the porcine TM tissue supports the house swine as an appropriate model system in terms of glaucoma TM proteomics. In conclusion, the present study provides a detailed view into the complex porcine TM proteome as an important reference for ocular studies, especially in terms of glaucoma research. It also represents an important contribution regarding “farm animal proteomics” [60], while strengthening the house swine as an attractive model organism in terms of ocular clinical proteomics. Also, the identification of neuronal-related proteins in the TM provides new input for the debate on TM origin and function.

## 4. Materials and Methods

### 4.1. Sample Preparation

Trabecular meshwork (TM) tissues were prepared from freshly enucleated eye bulbs (*n* = 30) from house swine *Sus scrofa domestica* individuals (sacrificed at 3–6 month, female: male = 3:2) provided by local slaughterhouses (Landmetzgerei Harth, Stadecken-Elsheim, Germany; Metzgerei Köppel, Mainz, Germany). The application of animal by-products for research purposes was approved by the Kreisverwaltung Mainz-Bingen in Germany (Identification Code: DE 07 315 0006 21, approved on 13 January 2014). Eye bulbs were equatorially opened with a scalpel removing lens, vitreous, iris and ciliary body. TM surrounding the porcine specific serial collector vessel “Schlemm‘s canal” representative [10] was carefully probed and isolated under a SZ 61 binocular microscope (Olympus, Tokyo, Japan), whereby tissue purity was visually inspected considering 5–40× magnification. TM tissue was immediately stored at −80 °C. TM tissues were homogenized by mechanical scratching using a 1.8 mm diamond milling head (LUX-TOOLS, Wermelskirchen, Germany), five freeze/thawing cycles (room temperature vs. −20 °C) followed by sonication. Homogenate aliquots corresponding to majority of eye bulbs (*n* = 24) were scheduled for one-step detergent protein extraction using separate detergents (detergent: homogenate = 1:1, *v*/*v*, (0.5% dodecyl-β-maltoside (DDM; Sigma-Aldrich, Steinheim, Germany), 0.1% sodium dodecyl sulfate (SDS; Sigma-Aldrich, Steinheim, Germany), 0.5% Tween 20 (Carl Roth, Karlsruhe, Germany), Triton-X-100 (Sigma-Aldrich, Steinheim, Germany)) and corresponding detergent mixture. Additionally sequential extraction was used, starting with 0.5% DDM followed by 0.1% SDS and finally 1% trifluoroacetic acid (TFA, Sigma-Aldrich, Steinheim, Germany) /20% acetonitrile (ACN; Applichem, Darmstadt, Germany). Additionally, TM samples from a few eye bulbs (*n* = 6) were extracted by use of two combinatory lysis buffers (buffer 1: 2M thiourea (Sigma-Aldrich, Steinheim, Germany), 7M urea (Carl Roth, Karlsruhe, Germany), 30mM Tris-HCl (Carl Roth, Karlsruhe, Germany) pH 8.5, 4% CHAPS (Sigma-Aldrich, Steinheim, Germany), 2% ASB14 (Sigma-Aldrich, Steinheim, Germany); buffer 2: 8mM Na_2_HPO_4_, 50mM Tris-HCl pH7, 137 mM natrium chloride (NaCl; Merck, Darmstadt, Germany), 3mM potassium chloride (KCl; Carl Roth, Karlsruhe, Germany), 0.5% SDS, 0.5% Triton-X-100, 0.5% DDM). Samples were centrifuged (2000× *g*, 15 min, 4°C) and extract supernatant aliquots were stored at −80°C for further BULCMS analysis.

### 4.2. Proteomic Analysis of TM Tissue

Protein concentration analysis of extracts was realized by use of the Pierce BCA protein assay kit (Thermo Fisher Scientific, Rockford, IL, USA) recorded by a Multiscan Ascent plate reader (Thermo Electron Corporation, Thermo Fisher Scientific, Rockford, IL, USA) at 570 nm according to the manufacturer‘s instructions. The TM samples (50 µg/sample) were run on 10-well NuPAGE 12% Bis-Tris minigels (Invitrogen, Carlsbad, CA, USA) for 1 h at 150 V under reduced conditions. Gels were fixed and stained using the Novex Colloidal Blue Staining Kit (Invitrogen, Carlsbad, CA, USA) according to the manufacturer‘s instructions followed by scanning on a DCP-9042 CDN bench top scanner (Brother Industries Ltd., Nagoya, Japan) at 1200 dpi. For tryptic in-gel digestion, a modified protocol of Shevchenkov and colleagues [61] was used. Solid phase extraction (SPE)-based peptide purification was carried out using C18 ZIPTIP pipette tips (Millipore, Billerica, MA, USA) according to the supplier’s protocol. For LC-MS/MS analysis, resolubilized (10 µL 0.1% TFA) peptides were fractionated using a Rheos Allegro (Thermo Fisher Scientific, Rockford, IL, USA) capillary HPLC system (BioBasic C18 pre (30 × 0.5 mm) & analytical column (150 × 0.5 mm); flow rate: 6.7 ± 0.03 μL/min) coupled to a LTQ Orbitrap XL system (Thermo Fisher Scientific, Rockford, IL, USA) with an electro spray ionization (ESI) source. For each peptide aliquot, an elution and MS record period of 50 min were set. The gradient program was set as follows: Buffers: A = 98% H2O, 1.94% ACN, 0.06% methanol, 0.05% formic acid; B = 95% ACN, 3% methanol, 2% H2O, 0.05% formic acid; gradient program: 15–20% B (0–2 min), 20–60% B (2–35 min), 60–100% B (35–40 min), 100–0% B (40–45 min), 0% B (45–50 min)]. MS parameters were set as follows: detection range= 300–2000 *m/z*, LTQ injection time = 50 ms, FT injection time = 500 ms, normalized energy of collision induced decay (CID) = 35, activation time = 30 ms, activation Q = 0.25, *m/z* isolation width for fragmentation = 2, dynamic exclusion = 90 s, repeat duration = 30 s, resolution = 30,000, centroid detection, fragmentation selection = top 5 monoisotopic *m/z* signals (*z* = 1+, 2+, 3+ and 4+, intensity >500). The BULCMS workflow was established in previous studies analyzing neuroretinal cell extracts [62,63] as well as human and porcine retinal tissues [21,52,64]. All resulting LC MS/MS Thermo RAW data were combined for protein identification using database-dependent protein identification in Proteome Discoverer (version 1.1, Thermo Fisher Scientific, Rockford, IL, USA) implementing the MASCOT search engine (version 2.2.07). A second protein identification search was performed using the combinatory database/de novo peptide sequencing software tool PEAKS (Studio version 7.5 trial; Bioinformatics Solutions Inc., Waterloo, ON, Canada). Mass tolerances were adjusted to 20 ppm (precursor peptides) and 0.8 Da (fragments). Carbamidomethylation (C) was set as fixed modification. Trypsin was chosen as enzyme and a maximum of two missed cleavages were allowed per peptide. For stringent protein identification, output was filtered confidently based on the false discovery rate (FDR) < 1%. In addition, for PEAKS output data, the matrix average local confidence peptide score (ALC) threshold was adjusted to >60%, and for proteins the −10logP score was set >20. For database screening, a combinatory strategy was selected considering *Sus scrofa* and *Homo sapiens* as taxonomies. A complemented TM protein list was scheduled for gene ontology (GO) analysis by use of Cytoscape (version 2.8.3 with BINGO 2.44 plugin; www.cytoscape.org). Thereby, GO categories “cellular component” and “molecular function” were screened for potential annotations. Moreover, the combined TM protein list was manually screened for functional protein associations. Protein-protein interaction (PPI) analysis was realized by use of STRING version 10 (http://string-db.org) [65]. For Venn chart analysis, the software package Venny (version 2.1, http://bioinfogp.cnb.csic.es/tools/venny/) [66] was used.

## Supplementary Materials

Supplementary materials can be found at https://www.mdpi.com/1422-0067/20/10/2526/s1. The supplementary file contains Tables S1 and S2. Identified proteins are listed in Table S1 and list of neuronal-related proteins is provided in Table S2.
